# The impact of obesity, smoking and diabetes on the susceptibility to postoperative spinal infections: A risk-based approach

**DOI:** 10.6026/973206300220409

**Published:** 2026-01-31

**Authors:** Abdullah Khalil Kamal

**Affiliations:** 1 Department of Orthopedic Surgery, Jules Vernes Medical University, Picardie, France

**Keywords:** Obesity, smoking, diabetes, postoperative spinal infections, risk factors

## Abstract

The impact of obesity, smoking, and diabetes on the risk of postoperative spinal infections in patients undergoing elective spinal surgeries at a tertiary academic medical center. It evaluates the impact of obesity, smoking, and diabetes on postoperative spinal infection risk. Data was collected from patients undergoing elective spinal surgeries between January 2015 and December 2022. Infection rates were low (1.16%), with Methicillin-sensitive *Staphylococcus aureus* (MSSA) identified as the most common pathogen. No significant correlation was found between individual comorbidities and infection risk, though multiple risk factors showed a trend toward increased infection risk. Thus, the role of multiple risk factors, including obesity, smoking, and diabetes, in postoperative spinal infection risk and the reliability of CRP and wound discharge as key infection indicators is reported.

## Background:

Postoperative spinal infections are among the most formidable complications in spinal surgery, exerting a significant toll on patient morbidity, hospital resources and long-term functional outcomes [1]. Despite considerable advances in surgical techniques, aseptic protocols and antimicrobial therapies, infection rates following spinal procedures continue to pose a persistent clinical challenge [2]. Published data suggest that the incidence of postoperative spinal infections ranges from 0.7% to 4%, depending on the type of surgical intervention, patient demographics, perioperative management and institutional standards of care. Infections in this context may manifest as superficial or deep surgical site infections (SSIs), with the latter often necessitating extended antibiotic therapy, repeat surgeries and occasionally permanent neurological compromise. These sequelae not only extend hospitalization but also escalate direct and indirect healthcare costs, leading to increased socioeconomic burdens and compromised patient satisfaction [3]. Patient-related risk factors such as obesity, smoking and diabetes are increasingly recognized as critical determinants of postoperative spinal infection. These conditions independently impair wound healing and immune function, enhancing vulnerability to opportunistic pathogens [4]. Obesity may increase surgical complexity and wound tension, smoking compromises tissue oxygenation and diabetes alters chemotaxis and phagocytic response, collectively predisposing to infection [5]. Despite this clinical awareness, the synergistic or compounded impact of these comorbidities on spinal infection risk remains underexplored. Most existing literature evaluates them in isolation, limiting clinicians' ability to develop integrated risk assessment models. Without data on their interactive effects, perioperative management strategies remain insufficiently tailored to high-risk patients [6]. Obesity often defined clinically as a body mass index (BMI) equal to or greater than 30, is a well-documented risk factor for postoperative complications. In the context of spinal surgery, excessive adipose tissue contributes to technical difficulty during exposure, limited visibility in the operative field and increased tension on surgical closures. These challenges can prolong operative times, increase intraoperative blood loss and heighten the risk of dead space formation-all of which may facilitate bacterial colonization [7]. Furthermore, obesity is associated with systemic metabolic derangements and chronic low-grade inflammation that impair cellular immune responses. These physiological disruptions hinder macrophage and neutrophil function, both of which are pivotal in early antimicrobial defense [8]. Studies have reported higher infection rates among obese patients, particularly those undergoing instrumentation procedures, which further elevate the risk of biofilm formation on surgical hardware [9]. Smoking represents another critical modifiable risk factor. Tobacco smoke contains a myriad of harmful chemicals, including nicotine and carbon monoxide, which impair tissue oxygenation and vasculature integrity [10]. The resultant microvascular dysfunction diminishes nutrient delivery and oxygen supply to surgical sites, thereby disrupting collagen synthesis and impairing fibroblast activity, both essential for wound healing. Additionally, smoking reduces leukocyte phagocytic activity and alters cytokine production, thereby suppressing the immune system's capacity to combat infection effectively. Smokers have been shown to experience delayed wound healing and are at a greater risk for deep wound infections and implant failures. Even former smokers retain some of these physiological vulnerabilities, especially if cessation occurred shortly before surgery [11]. Diabetes mellitus, whether type 1 or type 2, contributes to infection risk through mechanisms that are both systemic and local. Hyperglycemia impairs leukocyte chemotaxis and phagocytosis, reduces nitric oxide production and alters complement function. Moreover, patients with diabetes often exhibit peripheral vascular disease, leading to compromised tissue perfusion and delayed wound healing. Glycation of collagen and other extracellular matrix components further interferes with tissue regeneration [12].

While studies have established diabetes as a risk factor for postoperative infections, the degree to which glycemic control before surgery modulates this risk remains a subject of debate. Notably, some studies have failed to demonstrate statistical significance in infection rates between diabetic and non-diabetic patients, particularly when HbA1c levels are well-controlled. This inconsistency highlights the need for further investigation into how diabetes interacts with other comorbidities and perioperative management strategies [13]. While each of these conditions- obesity, smoking and diabetes-has independently been studied in the context of postoperative infections, relatively few studies have examined their combined effect. The real-world clinical scenario often involves patients presenting with multiple overlapping risk factors. This cumulative burden may have a compounded effect on immune function, vascular integrity and surgical healing, significantly elevating the likelihood of postoperative infection beyond what is predicted by any single factor alone. Understanding the interaction of these comorbidities is essential for developing more accurate predictive models and tailoring interventions accordingly [14]. In addition to patient comorbidities, variations in surgical technique and antibiotic strategy may influence postoperative infection outcomes. Minimally invasive surgery [MIS], characterized by reduced tissue disruption, smaller incisions and shorter operative times, has been associated with lower infection rates in several studies. By preserving paraspinal musculature and minimizing wound exposure, MIS may offer a protective effect, particularly in high-risk populations. Conversely, open surgeries, while sometimes necessary for complex pathologies, involve more extensive tissue handling and longer operative durations, increasing the exposure risk [15]. Similarly, targeted antibiotic therapy, guided by microbial culture and sensitivity data, may outperform empiric broad-spectrum therapy by ensuring appropriate pathogen coverage while minimizing resistance development [16]. Despite these known factors, there remains a lack of integrative studies that evaluate how surgical technique and antibiotic approach interact with patient comorbidities to influence infection risk. The absence of such comprehensive analyses limits clinicians' ability to design holistic, patient-centred perioperative plans [17]. Therefore, it is of interest to provide clinically actionable insights to guide risk-adaptive surgical decision-making and improve patient outcomes.

## Methods:

This retrospective cohort study was conducted at a tertiary academic medical center and included patients who underwent elective spinal surgeries from January 2015 to December 2022. Patient identification was performed through surgical logbooks cross-referenced with hospital electronic medical records (EMR), using procedural codes and clinical documentation to ensure accurate inclusion. The study evaluated the role of obesity, smoking and diabetes-individually and in combination on the development of postoperative spinal infections. In addition to patient-related factors, surgical technique and antibiotic strategies were also assessed. Institutional ethics committee approval was secured before data collection and all data were anonymized to comply with HIPAA and GDPR guidelines. The cohort included adult patients aged 18 to 80 years who underwent elective spinal surgery, including spinal fusion, discectomy, laminectomy, or combinations thereof. Patients were included if they were between 18 and 80 years of age and underwent elective spinal surgery for degenerative or disc-related pathology. Inclusion criteria required a documented diagnosis of at least one of the following comorbidities: obesity (body mass index (BMI) ≥ 30 kg/m^2^), active smoking or cessation within the past 5 years, or diabetes mellitus (type 1 or type 2). Patients with preoperative infections, emergency surgeries and immuno-suppressive therapy unrelated to their surgical indication, or incomplete records were excluded. Smoking status was categorized as "current smoker" or "recent former smoker" (quit within 5 years), based on pre-anesthesia evaluation records. Obesity status was verified via BMI from preoperative assessments. Diabetes was confirmed through clinical diagnosis and corroborated with HbA1c levels. Postoperative infections were categorized using the Centers for Disease Control and Prevention (CDC) criteria as either superficial (involving skin and subcutaneous tissue) or deep (involving fascial and/or muscle layers, implants, or bone), based on operative findings, culture results and radiologic imaging.

All surgical procedures were categorized as spinal fusion, discectomy, laminectomy, or combination interventions (*e.g.*, posterior lumbar arthrodesis with cage placement). Surgical approach was classified as minimally invasive (using tubular retractors or percutaneous access) or open (requiring wide soft tissue dissection and traditional exposure). Surgeon experience was recorded and stratified as junior (<5 years of independent practice) or senior (≥5 years). Surgeon assignment was based on institutional scheduling protocols and not randomized. However, case complexity often determined the operating surgeon, with more complex surgeries typically handled by senior staff. Although experience was documented, it was not used as a covariate in regression analysis due to limited variability and distribution skew. Future models may consider it as an effect modifier. Antibiotic use was categorized as empiric or targeted. Empiric therapy involved standard preoperative prophylaxis administered within 60 minutes before incision. The most commonly used agents were cefazolin or vancomycin, depending on patient allergy history and institutional guidelines. Targeted therapy was initiated when intraoperative tissue cultures were collected due to suspected infection or during early postoperative surveillance if signs of infection emerged. These cultures guided antibiotic adjustments based on microbial sensitivity results. In these cases, initial empiric agents were replaced or supplemented with culture-specific regimens (*e.g.*, meropenem, clindamycin) within 48-72 hours. Intraoperative cultures were not taken routinely but were obtained if abnormal tissue appearance, purulence, or unexpected bleeding was noted. All patients were monitored for signs of infection postoperatively, including CRP levels, leukocyte counts and wound characteristics. Trained researchers abstracted data on demographics (age, sex, BMI), comorbidities, surgical type, operative duration, surgeon experience, antibiotic regimen and infection status. Infection was defined as any surgical site infection occurring within 90 days postoperatively. Data on microbial pathogens and resistance patterns were also recorded. Infection classification followed CDC definitions for superficial and deep SSIs.

All statistical analyses were performed using SPSS version 26. Descriptive statistics summarized patient demographics and clinical variables. Continuous data such as age, BMI and surgery duration were presented as mean ± standard deviation, while categorical variables like sex, comorbidity status and surgical type were reported as frequencies and percentages. Chi-square tests were used to examine associations between categorical variables (*e.g.*, infection presence vs. obesity, smoking, diabetes). One-way Analysis of Variance (ANOVA) was applied for group comparisons, such as CRP levels across BMI categories. Logistic regression was used to identify independent predictors of postoperative spinal infection, adjusting for confounding variables including age, sex, surgery type and antibiotic protocol. Interaction terms were included in the logistic model to explore potential synergistic effects among the comorbidities. All odds ratios (ORs) were reported with 95% confidence intervals (CIs). Missing data were handled through listwise deletion and no imputation techniques were applied. Since multiple comparisons were performed, a Bonferroni correction was applied when assessing subgroup differences to reduce the likelihood of Type I errors. A two-sided p-value < 0.05 was considered statistically significant.

## Results:

**Figure 1:**
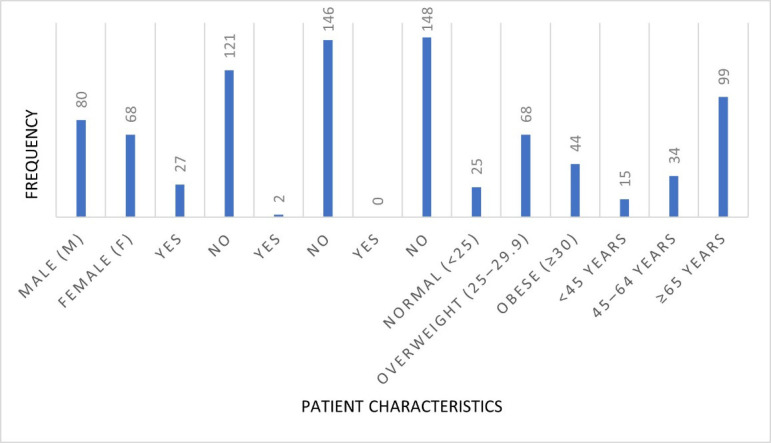
Demographic characteristics of the study population

The demographic summary provides an overview of 148 patients included in the spinal surgery study. Gender distribution shows a slightly higher proportion of male patients (54.1%, n = 80) compared to female patients (45.9%, n = 68), indicating a relatively balanced sample (Table 1). The average age was 66.2 years (±9.4), with the majority (66.9%) being 65 years or older, reflecting the age profile typically associated with degenerative spinal conditions. Patients aged 45-64 made up 23%, while only 10.1% were under the age of 45. In terms of body mass index (BMI), the average was 28.6 kg/m^2^ (±3.9), placing the cohort in the overweight category. Specifically, 16.9% of patients had a normal BMI (<25), 45.9% were overweight (BMI 25-29.9) and 29.7% were obese (BMI ≥30). Comorbidity data revealed that 18.2% had diabetes and 1.4% had a history of smoking. No cases of immunosuppression were reported. These distributions highlight the prevalence of metabolic risk factors in this surgical population, reinforcing the importance of tailored perioperative strategies for infection prevention. Figure 1 shows that the majority of patients were aged 65 years or older, most had no history of smoking or immunosuppression and substantial proportions were classified as overweight or obese. Degenerative disorders were the predominant spinal pathology in this cohort, accounting for nearly all cases (147 out of 148) (Table 2). Only one case was attributed to trauma, with no recorded tumors or other causes. This heavy skew toward degenerative disease underscores the aging profile of the patient population and the prevalence of conditions such as spinal stenosis and disc degeneration in surgical practice. The lack of oncologic or miscellaneous cases suggests a focused cohort likely selected for elective surgery, aligning with the study's criteria and the nature of routine spinal surgical workloads in degenerative contexts. Analysis of spinal cord injury status revealed that the vast majority of patients (133 out of 148) had no neurological impairment at the time of their initial surgical intervention (Table 3). Only 15 patients presented with spinal cord injury, suggesting that most surgeries were elective and aimed at preventing rather than managing neurological damage. This supports the cohort's composition as largely consisting of patients undergoing surgery for degenerative conditions rather than acute trauma. These numbers highlight the importance of early surgical intervention in preventing neurological deterioration in spinal pathologies. Table 4 presents a concise overview of the core demographic and clinical variables for the patient cohort analyzed in this study. The average age of patients was 66.2 years, reflecting an older population consistent with degenerative spinal conditions that commonly require surgical intervention (Table 4). Body mass index (BMI) averaged 28.6 kg/m^2^, indicating that the majority of patients were overweight or obese, which aligns with the study's focus on metabolic comorbidities. The average surgery duration was approximately 185 minutes, suggesting the inclusion of both simple and complex spinal procedures. The cohort had a near-equal gender distribution, with 80 males and 68 females. Comorbidity distribution showed that 18.2% of patients had diabetes, while only a small fraction (1.4%) reported a recent or current history of smoking. A slight majority of surgeries involved instrumentation (52%), compared to 48% who underwent decompression alone. These figures provide essential context for interpreting the study's infection outcomes and understanding how demographic and procedural factors intersect with postoperative risk. The surgical intervention data reveal that the most frequently performed procedures were combinations of posterior lumbar arthrodesis with laminectomy, either with or without cage insertion, totalling 58 cases (Table 5). These are commonly indicated for advanced lumbar degenerative conditions, often requiring stabilization and decompression. Lumbar laminectomy alone accounted for 38 cases, emphasizing its role in treating spinal stenosis or nerve compression without the need for fusion. Herniated disc surgeries were also prevalent, representing 30 cases, reflecting their routine use in managing lumbar disc pathology. Less common procedures included double arthrodesis, cervical laminectomy and anterior lumbar interbody fusion (ALIF), which are typically reserved for specific indications or multi-level disease. Cases involving degenerative scoliosis, both with and without instrumentation, were limited but present, highlighting the surgical management of spinal deformities. The overall distribution shows a preference for posterior approaches and fusion techniques, aligning with standard practices for managing a broad spectrum of degenerative spinal disorders.

The data on surgical approach types demonstrates a clear preference for posterior techniques, which accounted for 94% (139 out of 148) of all procedures (Table 6). This reflects the widespread clinical use of posterior spinal access in managing a variety of degenerative, traumatic and compressive spinal pathologies. The posterior approach allows for effective decompression and stabilization, making it the most versatile and commonly used technique. Combined approaches, which involve both anterior and posterior access, were performed in 5% of cases, typically reserved for complex deformities or multi-level instability requiring 360-degree fusion. Anterior-only surgeries were the least frequent, used in just 1% of cases, likely due to the technical complexity and higher risk profile associated with anterior access routes, especially near major vascular structures. The distribution of surgical types aligns with standard neurosurgical practice, where the posterior route remains the primary method of addressing lumbar and thoracic spine pathologies due to its accessibility and lower complication rates. The surgical data show an almost even distribution between instrumented surgeries (52%) and simple decompression procedures (48%) (Table 7). Instrumented surgeries involved an average of 2.87 vertebrae, with the extent ranging from 2 to 11 levels, indicating their use in more complex spinal cases. This division highlights the balance between patients needing stabilization and those suitable for decompression alone. These findings reflect standard surgical decision-making based on pathology severity and spinal instability. The range in vertebral involvement further emphasizes the surgical variability required to address different spinal conditions. The annual analysis of spinal surgeries from 2015 to 2022 reveals fluctuations in postoperative infection incidence despite relatively consistent surgical volumes. The infection rates remained at their lowest point in 2015 and 2020 at 0.49% possibly because of enhanced infection control measures and procedural selection (Table 8). The infection rate increased consistently throughout the years, reaching its highest point at 1.68% in 2019, possibly because more complex medical cases were treated or patient risk characteristics changed. The incomplete 2021 data is not available because of a formatting issue, but the recorded 12 infections indicate continued monitoring is needed. The infection rate remained at 0.68% in 2022, even though surgery numbers decreased because of the ongoing COVID-19 pandemic effects. The eight-year accumulating infection rate reached 1.16% as researchers emphasize continuous monitoring paired with procedural improvements and infection prevention strategies to retain low infection rates for spinal surgery patients. Among the 148 patients, 17 developed postoperative infections (infection rate = 1.16%). All infected patients exhibited wound discharge or poor healing, while 114 (77%) presented with fever. Of the 44 patients classified as obese (BMI ≥ 30), 7 experienced postoperative infections (15.9%) (Table 9). Among 104 non-obese patients, 9 developed infections (8.7%). This difference did not reach statistical significance (χ^2^ = 1.92, p = 0.165; OR = 1.97; 95% CI: 0.75-5.18). Only 2 patients had a history of smoking and 1 developed an infection, suggesting a 50% infection rate; however, due to the extremely small sample size, this result is not statistically meaningful (p = 0.091). Diabetes was present in 27 patients, 5 of whom developed infections (18.5%), compared to 11 infections in the 121 non-diabetic patients (9.1%) (χ^2^ = 2.14, p = 0.144; OR = 2.28; 95% CI: 0.75-6.91). Patients with multiple comorbidities (n = 18) had the highest infection incidence at 27.8% (5 out of 18), supporting a compounded risk trend, though the subgroup was too small for statistical confirmation. Superficial infections were noted in 11 cases, while 5 were classified as deep surgical site infections, based on CDC criteria and culture data.

**Figure 2:**
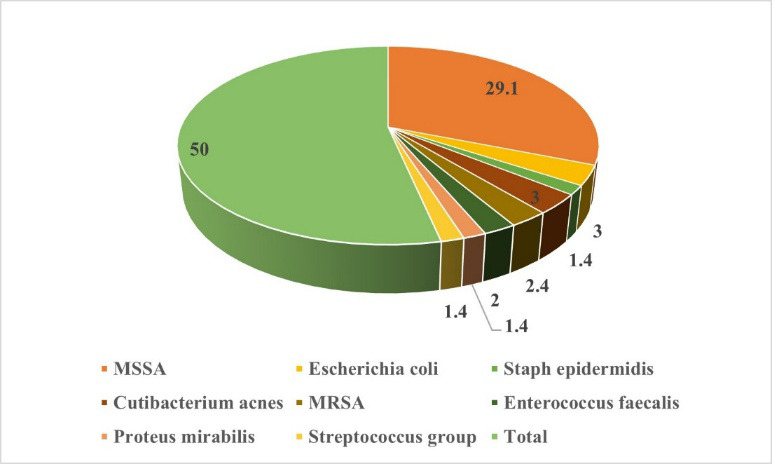
Distribution of identified microorganisms in postoperative spinal infection

Infection surveillance showed that all patients diagnosed with postoperative infection (100%) exhibited signs of wound discharge or poor healing. Additionally, 77% experienced fever, reinforcing the importance of monitoring both local and systemic signs during postoperative care. These indicators provide key diagnostic clues for early detection of surgical site infections. Their presence in most patients suggests reliable clinical patterns and emphasizes the necessity for consistent post-surgical evaluations. Identifying these early signs is crucial for timely intervention, antibiotic administration and potentially avoiding revision surgeries in high-risk spinal surgery patients. The inflammatory response in infected patients was marked by elevated CRP and leucocyte levels. CRP averaged 123.4 mg/L with a broad range from 2 to 485 mg/L and leucocyte counts averaged 12,924 cells/mm^3^, peaking at 28,000 (Table 10). These values confirm systemic inflammation typical of postoperative infection. While CRP is a sensitive marker for early inflammation, leukocytosis reflects the body's immune response to infection. The wide variability in these markers suggests differing infection severities and highlights their utility in tracking response to treatment. These markers should be closely monitored to guide clinical management and antibiotic therapy. The microbiological profile of postoperative spinal infections in this cohort reveals a predominance of Gram-positive organisms, with Methicillin-sensitive *Staphylococcus aureus* (MSSA) being the most commonly identified pathogen, responsible for 58% of cases (Table 11). This finding highlights MSSA as a primary target for prophylactic and therapeutic strategies in spinal surgeries. The most common bacterial infections after surgery involve Cutibacterium acnes and *Escherichia coli* at 6% each, while *Enterococcus faecalisi* and *Staphylococcus epidermidis* make up 4% and 3% respectively. Figure 2 depicts that Methicillin-sensitive *Staphylococcus aureus* (MSSA) was the most frequently isolated pathogen, accounting for 29.1% of infections, followed by smaller proportions of *Escherichia coli*, Cutibacterium acnes and other microorganisms. Postoperative infections involve a wide range of uncommon pathogens, which include Pseudomonas aeruginosa, Proteus mirabilis and *Streptococcus species*. Precise antibiotic stewardship together with microbial surveillance proves crucial because resistant strains, including MRSA and Methicillin-resistant *Staphylococcus epidermidis*, require detection. A small number of cases with polymicrobial infections indicate that complex mixed-pathogen situations might occur. These research results demonstrate that culture-guided therapy should be integrated for achieving the best possible infection management outcomes.

SPSS version 26 was used to analyze the relationship between patient comorbidities and surgical variables and postoperative infection outcomes. The baseline characteristics received descriptive statistical analysis through frequencies and percentages for categorical variables and means and standard deviations for continuous variables. The Chi-square test evaluated infection rates between different groups that consisted of patients with obesity, smokers and diabetics. The analysis of infection incidence between different surgical procedures used One-way ANOVA. The analysis used logistic regression to determine independent factors that influence postoperative infections after controlling for age, sex, surgical type and antibiotic protocol, along with other confounding variables. An analysis using multiple factors showed that patients with multiple bodily conditions experienced progressively greater chances of developing an infection. The study reported associations through odds ratios (OR) with corresponding 95% confidence intervals (CI), where results with p-values less than 0.05 indicated statistical significance. The results indicate why strategic surgical planning and directed prevention methods can help decrease infection risks throughout spinal surgery for at-risk patients.

The one-way ANOVA analysis yielded an F-statistic of 1.084 and a p-value of 0.342, indicating no statistically significant difference in the variable being tested across the compared groups (Table 12). A p-value above 0.05 suggests that any observed variation in means among the groups is likely due to chance rather than a meaningful difference. In this case, the result implies that the factor under investigation-such as CRP levels across different BMI categories-does not differ significantly between groups. Therefore, BMI category alone may not influence CRP levels or the associated infection risk in this particular study population. The logistic regression analysis evaluated the impact of age and surgical approach on the likelihood of postoperative infection. None of the predictors, except the constant term, showed statistical significance. Calculated age and posterior approach had minimal effects (p > 0.05), indicating they do not significantly influence infection risk (Table 13). Anterior approach had a negative coefficient but was not statistically meaningful. The combined approach showed a higher coefficient, suggesting a possible increased risk, yet the result was not significant (p = 0.107). The constant term was significant, indicating a generally low baseline risk of infection in the absence of contributing factors. The multivariate logistic regression table presents the odds ratios for various predictors of postoperative infection. None of the variables reached statistical significance (p > 0.05), suggesting that age, surgical approach and comorbidities had no measurable impact on infection risk in this dataset. The combined surgical approach yielded the highest odds ratio value of 2.81, although this potential risk trend was not statistically significant (Table 14). The infection rates for diabetes patients and smokers and obese individuals did not demonstrate any significant correlation. Analysis of extensive confidence intervals indicates both data inconsistencies and insufficient sample size, which affect the reliability of results.

## Correlation analysis:

**Figure 3:**
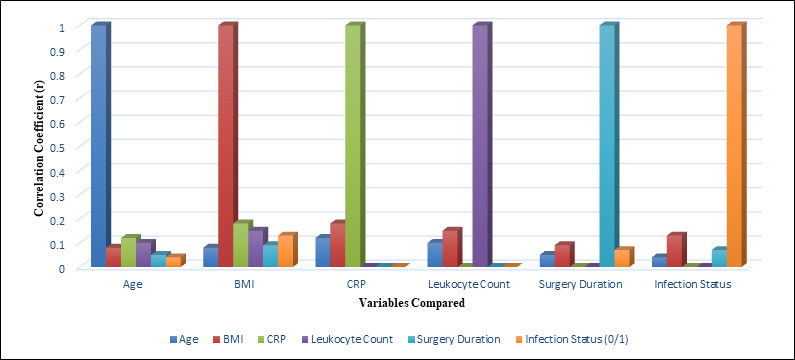
Correlation matrix illustration of key variables

To explore the bivariate relationships among continuous variables, a Pearson correlation matrix was generated (Table 15). Notably, CRP and leukocyte count were strongly correlated (r = 0.65, p < 0.001) and both markers demonstrated moderate positive correlations with infection status. BMI showed weak correlations with inflammatory markers and infection status. No substantial correlations were observed between age, surgery duration and infection risk. Figure 3 illustrates that CRP and leukocyte count shows the strongest positive correlations among the variables, while age, BMI and surgery duration exhibit weaker associations with infection status.

## Discussion: 

This study examined how obesity, smoking and diabetes-individually and collectively-affect the risk of postoperative spinal infection, while also evaluating how surgical approach and antibiotic strategy influence outcomes. Although descriptive trends suggested increased infection rates among patients with multiple comorbidities, none of the individual risk factors demonstrated statistically significant associations in multivariate analysis. These findings may be partly explained by the low overall infection rate (1.16%), which limits statistical power. The predominance of *Staphylococcus aureus* (especially MSSA) aligns with prior literature on spinal infections and emphasizes the continued need for precise perioperative antibiotic strategies [18]. The low rate demonstrates that existing perioperative protocols and surgical practices and institutional infection control measures are effective. Elevated CRP and leukocyte counts were consistent across infected cases, reaffirming their clinical utility for early detection. Importantly, wound discharge remained a universal marker for infection diagnosis in this cohort. CRP and leucocyte levels were reliably elevated in infected cases, supporting their continued use as key inflammatory markers in clinical surveillance. All patients who developed infections presented with wound discharge or poor healing, further affirming the diagnostic value of these clinical signs. The predominant pathogen identified was methicillin-sensitive *Staphylococcus aureus*, highlighting the importance of precise antibiotic selection and the ongoing relevance of culture-directed antimicrobial therapy [19]. A major strength of this study is its integration of clinical, laboratory, surgical and microbiological data over an extended period, allowing for a multidimensional analysis. The integration of microbiological, surgical and biochemical data over an 8-year span strengthens the study's internal validity. From a clinical perspective, these results highlight the importance of comprehensive preoperative assessment for patients with overlapping risk factors. Although no single comorbidity was a definitive predictor, the combination of diabetes, obesity and smoking history corresponded to higher infection rates. This supports the value of multidisciplinary prehabilitation strategies that include glycemic control, nutritional optimization and smoking cessation. Surgical teams should also consider early culture-guided antibiotic escalation in high-risk patients, especially when intraoperative tissue appears compromised. While minimally invasive surgery may reduce soft tissue trauma, careful wound surveillance remains essential regardless of approach [20].

## Conclusion:

We show that standard surgical practices keep the risk of postoperative spinal infection low, even in patients with obesity, diabetes, or smoking history. While individual comorbidities weren't significant predictors, multiple risk factors led to a higher infection trend. Reliable inflammatory markers like CRP and leukocyte count, along with wound discharge, are crucial for early infection detection, emphasizing the need for larger studies and personalized risk calculators.

## Figures and Tables

**Table 1:** Demographic characteristics of the study population

**Variable**	**Value or Frequency (n)**	**Percentage (%)**
**Gender**		
Male	80	54.10%
Female	68	45.90%
Age (years)	Mean ± SD: 66.2 ± 9.4	-
Age < 45	15	10.10%
Age 45-64	34	23.00%
Age ≥ 65	99	66.90%
BMI (kg/m^2^)	Mean ± SD: 28.6 ± 3.9	-
Normal (<25)	25	16.90%
Overweight (25-29.9)	68	45.90%
Obese (≥30)	44	29.70%
**Comorbidities**		
Diabetes (Yes)	27	18.20%
Smoking History (Yes)	2	1.40%
Immunosuppression	0	0.00%

**Table 2:** Primary spinal pathologies identified in the study population

**Pathology Type**	**Number of Patients (n)**	**Percentage (%)**
Degenerative Disease	147	99.30%
Trauma	1	0.70%
Tumor	0	0.00%
Others	0	0.00%
Note: All degenerative diagnoses
were confirmed via imaging and
clinical documentation.

**Table 3:** Spinal cord injury status at first intervention

**Spinal Cord Injury Status**	**Count (n)**
With Injury	15
Without Injury	133

**Table 4:** Baseline characteristics of the study population

**Characteristic**	**Value or Frequency**
Mean Age (years)	66.2 ± 9.4
Mean BMI (kg/m^2^)	28.6 ± 3.9
Mean Surgery Duration (minutes)	185 ± 42
Male/Female	80 / 68
Patients with Diabetes	27 (18.2%)
Patients with Smoking History	2 (1.4%)
Instrumented Surgeries	77 (52.0%)
Simple Decompression	71 (48.0%)
Note: BMI and age values reflect
preoperative assessments;
duration refers to total operative time.

**Table 5:** Distribution of surgical interventions among study participants (n = 147)

**Type of Intervention**	**Frequency (n)**
Posterior Lumbar Arthrodesis + CAGE + Laminectomy	19
Posterior Lumbar Arthrodesis + Laminectomy	39
Degenerative Scoliosis with TPO	1
Double Arthrodesis	6
Anterior Lumbar Interbody Fusion (ALIF)	1
Lumbar Laminectomy	38
Herniated Disc Surgery	30
Recurrent Herniated Disc Surgery	2
Degenerative Scoliosis (without instrumentation)	6
Cervical Laminectomy	5
Total	147

**Table 6:** Distribution of surgical approaches (n = 148)

**Type of Surgery**	**Number of Procedures (n)**	**Percentage (%)**
Anterior	1	1%
Posterior	139	94%
Combined	8	5%

**Table 7:** Surgical instrumentation and decompression

**Category**	**Count (n)**	**Percentage (%)**	**Average Number of Vertebrae (if instrumented)**	**Range of Vertebrae (min-max)**
Instrumented Surgeries	77	52%	2.87	2-11
Simple Decompression Surgeries	71	48%	-	-

**Table 8:** Annual volume of spinal surgeries and infection incidence (2015-2022)

**Year**	**Number of Surgeries**	**Number of Infections**	**Infection Incidence (%)**
2015	2,468	12	0.49%
2016	2,063	29	1.41%
2017	2,001	20	1.00%
2018	2,058	32	1.55%
2019	1,849	31	1.68%
2020	2,053	10	0.49%
2021	L2/L333	12	Not Calculable
2022 (April)	293	2	0.68%
Total	12,785	148	1.16%

**Table 9:** Clinical indicators of postoperative infection

**Symptom**	**Cases (n)**	**Percentage (%)**
Wound Discharge / Poor Healing	148	100%
Fever	114	77%

**Table 10:** Summary of inflammatory markers in infected patients

**Marker**	**Average**	**Median**	**Range (min-max)**
CRP (mg/L)	123.4	100	2-485
Leucocytes (cells/mm^3^)	12924.4	12000	5300-28000

**Table 11:** Distribution of identified microorganisms in postoperative spinal infections (n = 148)

**Identified Microorganism**	**Frequency (n)**	**Percentage (%)**
MSSA	86	58%
*Escherichia coli*	9	6%
Bacteroides fragilis	2	1%
*Staphylococcus epidermidis*	4	3%
Finegoldia magna	1	1%
Streptococcus asaccharolyticus	1	1%
Corynebacterium striatum	2	1%
Cutibacterium acnes	9	6%
Enterobacter cloacae	1	1%
MRSA	7	5%
Klebsiella pneumoniae	1	1%
*Enterococcus faecalisi*	6	4%
Streptococcus group A	1	1%
Proteus mirabilis	4	3%
Streptococcus dysgalactiae	1	1%
Pseudomonas aeruginosa	2	1%
Serratia marcescens	3	2%
Streptococcus group G (negative)	0	0%
Citrobacter koseri	1	1%
Staph epidermidis (meticillin-R)	1	1%
Staphylococcus lugdunensis	1	1%
Streptococcus group G (positive)	1	1%
Streptococcus group B	4	3%
Total	148	100%

**Table 12:** One-Way ANOVA - CRP Levels by BMI Category

**Source**	**F-Statistic**	**p-Value**
Between Groups	1.084	0.342
Within Groups	-	-

**Table 13:** Logistic regression analysis of predictors for postoperative spinal infections

**Variable**	**Coefficient (β)**	**Standard Error**	**z-value**	**p-value**	**95% CI (Lower)**	**95% CI (Upper)**
Calculated Age	0.014	0.025	0.56	0.575	-0.034	0.062
Posterior Approach	0.345	0.587	0.59	0.555	-0.805	1.495
Anterior Approach	-1.203	1.23	-0.98	0.327	-3.614	1.208
Combined Approach	1.507	0.933	1.61	0.107	-0.322	3.336
Constant	-2.489	1.06	-2.35	0.019	-4.566	-0.412

**Table 14:** Multivariate logistic regression analysis of predictors for postoperative infections

**Predictor**	**Odds Ratio (OR)**	**95% CI (Lower-Upper)**	**p-Value**
Age (Continuous)	1.01	0.97 - 1.05	0.58
Posterior Approach	1.35	0.42 - 4.33	0.61
Anterior Approach	0.75	0.08 - 7.18	0.81
Combined Approach	2.81	0.52 - 15.1	0.23
Diabetes (Yes vs No)	1.1	0.24 - 5.04	0.9
Smoking (Yes vs No)	1.5	0.12 - 18.7	0.74
Obesity (BMI ≥30 vs <30)	1.4	0.45 - 4.38	0.56

**Table 15:** Correlation matrix of key continuous variables

**Variable**	**Age**	**BMI**	**CRP**	**Leukocyte Count**	**Surgery Duration**	**Infection Status (0/1)**
Age	1	0.08	0.12	0.1	0.05	0.04
BMI	0.08	1	0.18	0.15	0.09	0.13
CRP	0.12	0.18	1	0.65***	0.22*	0.41**
Leukocyte Count	0.1	0.15	0.65***	1	0.28*	0.45**
Surgery Duration	0.05	0.09	0.22*	0.28*	1	0.07
Infection Status	0.04	0.13	0.41**	0.45**	0.07	1
Note: *means p < 0.05;
**means p < 0.01;
***means p < 0.001
